# Fractional CO_2_ laser versus 1064-nm long-pulsed Nd:YAG laser for inflammatory acne vulgaris treatment: a randomized clinical trial

**DOI:** 10.1007/s10103-023-03855-6

**Published:** 2023-08-18

**Authors:** Tasneem Muhammad Hammoda, Naglaa Abdallah Ahmed, Mervat Hamdino

**Affiliations:** Dermatology and Venereology Department, Faculty of Medicine for Girls, Al-Azhar University, Cairo, Egypt

**Keywords:** Fractional CO_2_ laser, Long-pulsed Nd:YAG laser, Inflammatory acne vulgaris

## Abstract

Acne vulgaris is challenging to treat for several individuals. Laser therapy may be a desirable alternative to traditional therapies with limited success. This study aimed to assess efficacy of fractional CO_2_ laser versus Nd:YAG laser for acne vulgaris therapy. Thirty cases with acne vulgaris underwent both fractional CO_2_ laser and Nd: YAG laser treatments in a randomized split face design at a 14-day interval for four sessions. The clinical efficacy was evaluated by counting acne lesions and utilizing the Global Acne Severity Scale (GEA Scale). GEAs decreased significantly after both fractional CO_2_ and Nd:YAG modalities after treatment and at a 3-month follow-up; fractional CO_2_ demonstrated significant more decrease in GEAs with (*P* = 0.006, 0.00 (respectively. Moreover, fractional CO_2_ showed a significantly higher satisfaction level (*P* = 0.004) and a better clinical improvement percentage regarding inflammatory and noninflammatory acne lesions (*P* = 0.007 and 0.000, respectively) after 3 months of follow-up. Apart from transient erythema, there were insignificant adverse effects concerning both treated sides. Fractional CO_2_ and Nd:YAG lasers are efficient physical modalities of acne treatment. However, fractional CO_2_ laser was more effective and more satisfying to the patients.

## Introduction

Acne vulgaris is one of the main reasons for dermatological consultation with substantial physical and psychosocial burden [1]. It ranked 8th in overall disease prevalence worldwide [2]. About 43–69% of acne patients have acne scars, negatively impacting self-confidence and self-esteem [3]. Therefore, to avoid acne’s potential long-term complications, it is vital to take measures beyond lowering the number of lesions [4].

Numerous laser devices were proven successful in acne therapy, as they provide an efficient treatment of acne with a short recovery period and fewer drawbacks, notably the Nd:YAG laser, which has been documented with several studies [5, 6].

The mechanism of action of 1064-nm Nd:YAG laser relies on its action on the vascular element of inflammatory acne together with the alteration of cytokine release. While the improvement of non-inflammatory lesions can be attributed to the thermal damage to sebaceous glands causing sebum reduction [5].

Fractional CO2 laser has been established as one of the best options of acne scarring therapy. Although, it can be hypothesized that fractional CO2 ablative laser can be a proper modality for inflammatory acne vulgaris, based on its effects on the various mechanisms of acne production [7].

As, fractional CO_2_ lasers generate sebaceous gland photothermolysis, by causing tissue ablation zones across a portion of the dermis and epidermis with nearby tissue coagulation. So, it can be promising in acne vulgaris treatment as sebaceous gland is the site involved in acne production [8, 9].

There is limited evidence and few studies about benefits of fractional CO2 lasers for treating inflammatory acne. Consequently, the current trial aimed to asses efficacy and safety of fractional CO_2_ laser against 1064-nm long-pulsed Nd:YAG laser which has been established by many studies in acne vulgaris.

## Material and methods

This prospective randomized split-face comparative study was conducted with 30 patients with acne vulgaris. Patients were selected from the attendants of the Dermatology Outpatient Clinic of Al Zahraa University Hospital, Cairo, Egypt.

The study was performed over a period from July 2021 to September 2022. The study was approved by the Research Ethics Committee of Faculty of Medicine for Girls, Al-Azhar University, Cairo, Egypt with approval code (2021121145) and following the Helsinki Declaration. Written informed consent was obtained from all participating patients.

Inclusion criteria included adult patients > 18 years old experiencing mild to severe acne according to the Global Acne Severity Scale (GEA Scale) [10].

Exclusion criteria included age below 18, pregnancy or lactation, systemic diseases, a background of hypertrophic scars or keloids, photosensitivity, history of herpes infection, active infection, dermatitis, malignancy over the treatment area, hormone replacement therapy, patients taking contraceptive pills, topical acne therapies or systemic antibacterial agents 30 days preceding the study, or oral retinoid 6 months prior to enrollment; additionally, individuals with mild comedonal acne or nodulocystic acne were excluded from the trial.

All patients provided a thorough medical history and had a comprehensive general and dermatological assessment and pretreatment photographs. Subjective clinical assessment, lesions counting [11], and grading of severity by GEA scale [10] were done by two blinded independent dermatologists.

### Treatment sessions

Each patient received four laser treatment sessions at 14 days of interval: Nd:YAG laser on half of the face and fractional CO_2_ on the other half; each half was randomly determined by selecting an enclosed opaque envelope with a card labeled with Nd:YAG or fractional CO_2_ laser, taking into account the therapy for the left and right split-face sides.

Before the session, the face was washed with water, and an anesthetic cream (Pridocaine cream®; lidocaine 2.5% and prilocaine 2.5%) was left for 40 min. After removing it, skin was cleansed with 70% alcohol.

On one side, a long-pulsed Nd:YAG 1064-nm laser (DEKA, Synchro FT, Italy) was utilized. Lesions were treated with three consecutive overlapped passes, while the perilesional region (approximately 2 cm around the lesion) and the unaffected region (approximately 10 cm around the lesion) were treated with one pass. The fluence was 35 J/cm^2^ for the lesion and 30 J/cm^2^ for nearby and unaffected tissue; the pulse frequency was 20 ms; the spot size was 10 mm; and the cooling tip temperature was 4 °C.

On the opposite side, fractional CO_2_ 10,600-nm laser (DEKA, SmartXide DOT, Italy) was applied. The lesions were treated with two passes that overlapped, whereas the perilesional and uninvolved regions were medicated with a single pass. A power of 15 watts, dot mode with a spacing of 550 m, dwell time of 400 s, smart track, scanning mode, single stack, square form, a ratio of 10/10, and size of 100%, were the therapy parameters with fine adjustment of these parameters according to the patient’s skin type and reaction. These settings resulted in a density of 11.9%, a fluence of 0.74 J/cm^2^, and an energy/dot of 6 mJ.

Ice packs were immediately applied to both sides after laser treatment. Texacort 0.1% (Hydrocortisone 17- butyrate 1 mg/gm) was advised for 3 days after the session to reduce post laser inflammation. Patients were instructed to avoid sun exposure and using cosmetics or any topical or systemic medications during the study period and to apply sunscreen with SPF 50 on their whole face every day.

### Efficacy assessments

Primary assessment was done by count of acne lesions at 1 month after the last session [12, 13].

Secondary assessment was performed through severity grading by the Global Acne Severity Scale (GEA Scale), acne lesions improvement percentage, and patient’s satisfaction [12, 13].

Clinical images were taken by an iPhone 6 s Plus (12 mega pixel, f/2.2 mm, LED flash, China) at baseline, each session, 1 month after the last session, and a 3-month follow-up after the last treatment.

Acne lesions counting [11] and severity grading by GEA Scale [10] were conducted by two blinded independent dermatologists.

Patient Global Impression of Change (PGIC), a 7-point scale representing a patient’s rating of overall improvement was conducted [14].

Pain level was evaluated by the patients after each treatment via a 10-point visual analog scale (VAS 1 = no pain, 10 = worst pain ever felt).

The degree of clinical improvement of facial pore size was recorded according to the Quartile improvement scale [15].

Any changes in scar grading were documented using the Goodman and Baron qualitative and quantitative acne scarring grading system [16, 17].

The expected adverse effects are the following: (a) Erythema that lasts longer than 48 h which is graded on a three-point scale (at level of 0; “no erythema,” at level of 3; “severe erythema”). (b) Post laser hyperpigmentation (a score of 0 indicates no hyperpigmentation following laser therapy, whereas a score of 1 indicates the existence of hyperpigmentation). (c) Post laser hypopigmentation (a score of 0 shows the lack of hypopigmentation after laser treatment, whereas a value of 1 indicates the existence of hypopigmentation).

Recurrence was defined as appearance of same lesions after the whole therapy or the development of novel lesions. Patients’ satisfaction levels were assessed through the VAS (0–10; 0: “not satisfied,” 10: “completely satisfied”).

### Statistical analysis

Data were managed through the Statistical Package for Social Science (IBM SPSS) version 23. The comparison between two modalities of treatment with qualitative data was done by using Chi-square test. The comparison between two modalities of treatment with quantitative data and non-parametric distribution was created by using Mann–Whitney test and Willcoxon test. For all previous tests, *P* value < 0.05 was set as statistically significant.

## Results

The study involved 30 female cases, aged 18 to 24 years, with a mean ± SD of 21 ± 1.62. Eight cases were skin phototype IV, and 22 participants were Fitzpatrick skin type III.

The comparison of both sides revealed that after treatment (4 weeks after the final session), a statistically significant reduction in mean inflammatory count in the fractional CO_2_ side compared to the Nd:YAG side (*P* = 0.044) was detected, but no statistically significant difference was found in mean noninflammatory acne count between both therapeutic modalities (*P* = 0.054). But, after 3 months’ follow-up, fractional CO_2_ evoked a statistically significant reduction in mean inflammatory and noninflammatory lesions compared with Nd:YAG (with *P* values of 0.003, 0.004, respectively) (Table 1).

**Table 1:** Comparison between fractional side and Nd-YAG side regarding inflammatory and non-inflammatory lesions count at baseline, after treatment, and at a 3-month follow-up

Parameters		Fractional side	Nd-YAG side	Test value‡	*P* value
		No. = 30	No. = 30		
Inflammatory count					
Baseline	Mean ± SD Median (IQR) Range	15.77 ± 8.89 13.5 (8 – 23) 4 – 31	14.07 ± 6.09 14 (8 – 19) 3 – 25	−0.363	0.717
After sessions	Mean ± SD Median (IQR) Range	1.53 ± 1.50 1 (0 – 3) 0 – 6	2.67 ± 2.35 3 (1 – 4) 0 – 10	−2.016	0.044
After 3 months of follow-up	Mean ± SD Median (IQR) Range	1.50 ± 1.17 1.5 (1 – 2) 0 – 5	3.17 ± 2.31 3 (1 – 5) 0 – 8	−2.943	0.003
Non-inflammatory count					
Baseline	Mean ± SD Median (IQR) Range	36.17 ± 24.1 30 (15 – 55) 3 – 88	30.97 ± 25.07 19 (14 – 45) 4 – 97	−0.969	0.333
After sessions (1 month)	Mean ± SD Median (IQR) Range	6.47 ± 5.31 7 (3 – 9) 0 – 20	11.10 ± 9.71 7.5 (5 – 14) 1 – 35	−1.928	0.054
After 3 months of follow-up	Mean ± SD Median (IQR) Range	3.77 ± 2.78 3 (2 – 6) 0 – 11	8.63 ± 7.53 6 (3 – 12) 2 – 27	−2.901	0.004

*P* value > 0.05: non significant (NS); *P* value < 0.05: significant (S); *P* value < 0.01: highly significant (HS)

^‡^: Mann–Whitney test

**Fig. 1:**
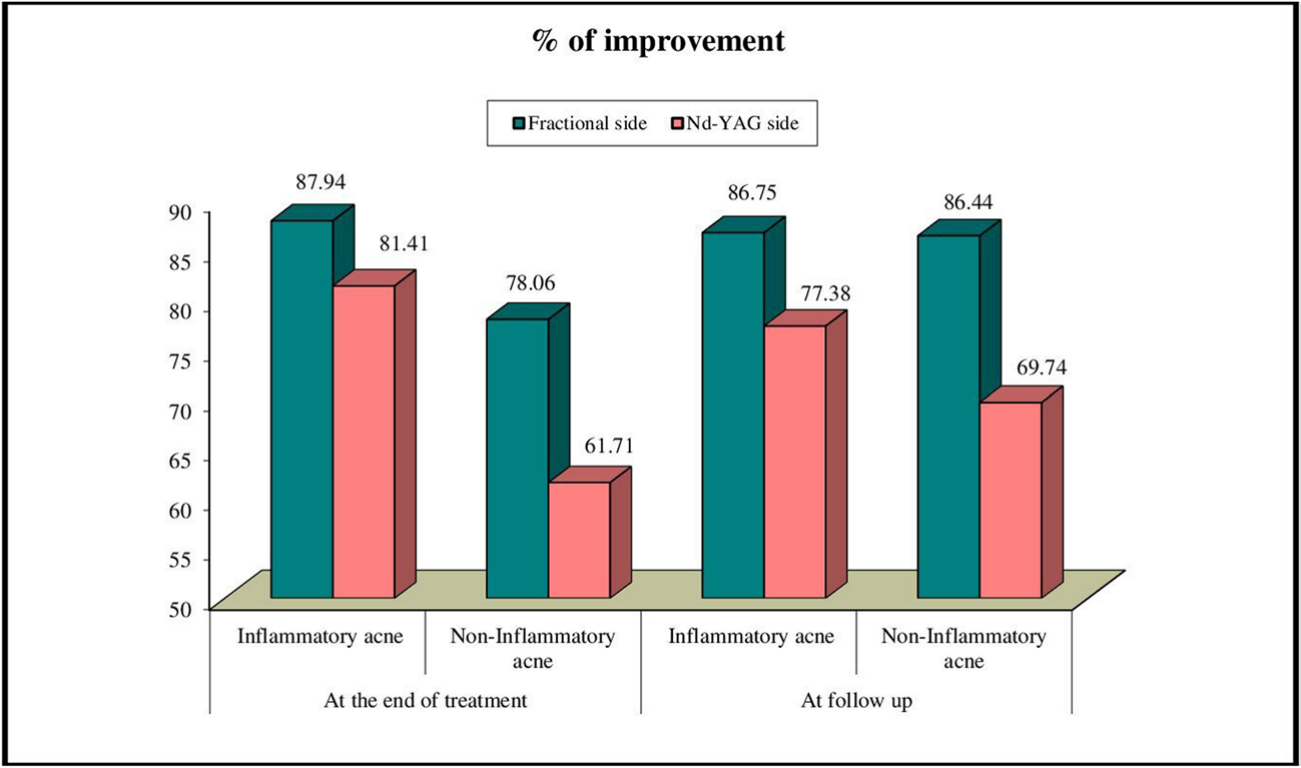
Comparison between fractional side and Nd: YAG side regarding inflammatory and non-inflammatory acne improvement percentage at treatment end and after follow up

The improvement percentage of inflammatory lesions at the fractional laser side was (mean ± SD = 87.94% ± 14.31) after treatment and reduced to (mean ± SD = 86.75% ± 12.67) following 3 months of follow-up. The noninflammatory acne improvement percentage at the fractional laser side was (mean SD = 78.06% ± 19.51) after treatment and increased to (mean ± SD = 86.44% ± 17.81) following 3 months of follow-up (Fig. 1).

Inflammatory acne’s improvement percent at Nd:YAG treated side was (mean ± SD = 81.41% ± 16.54) after treatment and reduced to (mean ± SD = 77.38% ± 15.08) following 3 months of follow-up, while improvement percentage of noninflammatory acne was (mean ± SD = 61.71% ± 21.22) after treatment, and elevated to (mean ± SD = 69.74% ± 14.13) following 3 months of follow-up (Fig. 1).

Furthermore, GEAs showed a statistically significant reduction by two modalities, following the end of therapy and after 3 months of follow-up. The fractional laser-treated side demonstrated better results (*P* = 0.006 and 0.002, respectively) (Table 2).

**Table 2:** Comparison between fractional side and Nd-YAG side regarding GEAs at baseline, after treatment, and at a 3-month follow-up

GEA		Fractional side	Nd-YAG side	Test value ‡	*P* value
		No. = 30	No. = 30		
Base line	Mean ± SD Median (IQR) Range	3.43 ± 0.86 3 (3 – 4) 2 – 5	3.40 ± 0.89 3 (3 – 4) 2 – 5	−0.166	0.868
After sessions	Mean ± SD Median (IQR) Range	0.87 ± 0.73 1 (0 – 1) 0 – 2	1.47 ± 0.82 1 (1 – 2) 0 – 3	−2.733	0.006
After a 3-month follow-up	Mean ± SD Median (IQR) Range	0.47 ± 0.63 0 (0 – 1) 0 – 2	1.17 ± 0.91 1 (0 – 2) 0 – 3	−3.107	0.002

*P* value > 0.05: non significant (NS); *P* value < 0.05: significant (S); *P* value < 0.01: highly significant (HS)

^‡^: Mann–Whitney test

**Fig. 2:**
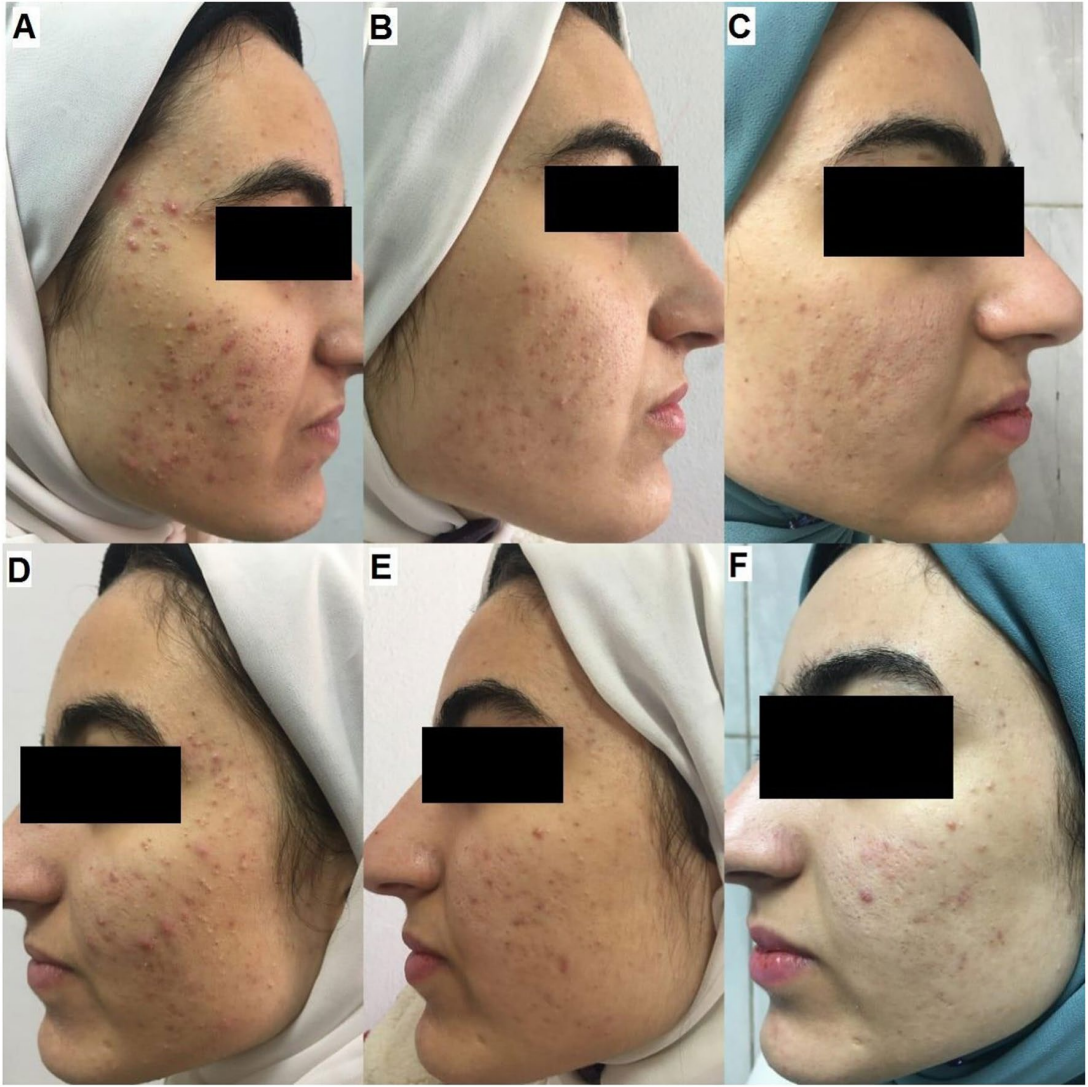
A 20-year-old-female patient with sever inflammatory acne (GEA scale was 4) received four treatment sessions. **A** Fractional laser side before treatment (GEAs 4, severe). **B** Fractional side at the end of treatment (1 month) (GEAs 1, almost clear). **C** Fractional side at a 3-month follow-up (GEAs 0, clear). **D** Nd-Yag laser side before treatment (GEAs 4, severe). **E** Nd-Yag side at the end of treatment (GEAs 2, mild). **F** Nd-Yag side at a 3-month follow-up (GEAs 2, mild)

**Fig. 3:**
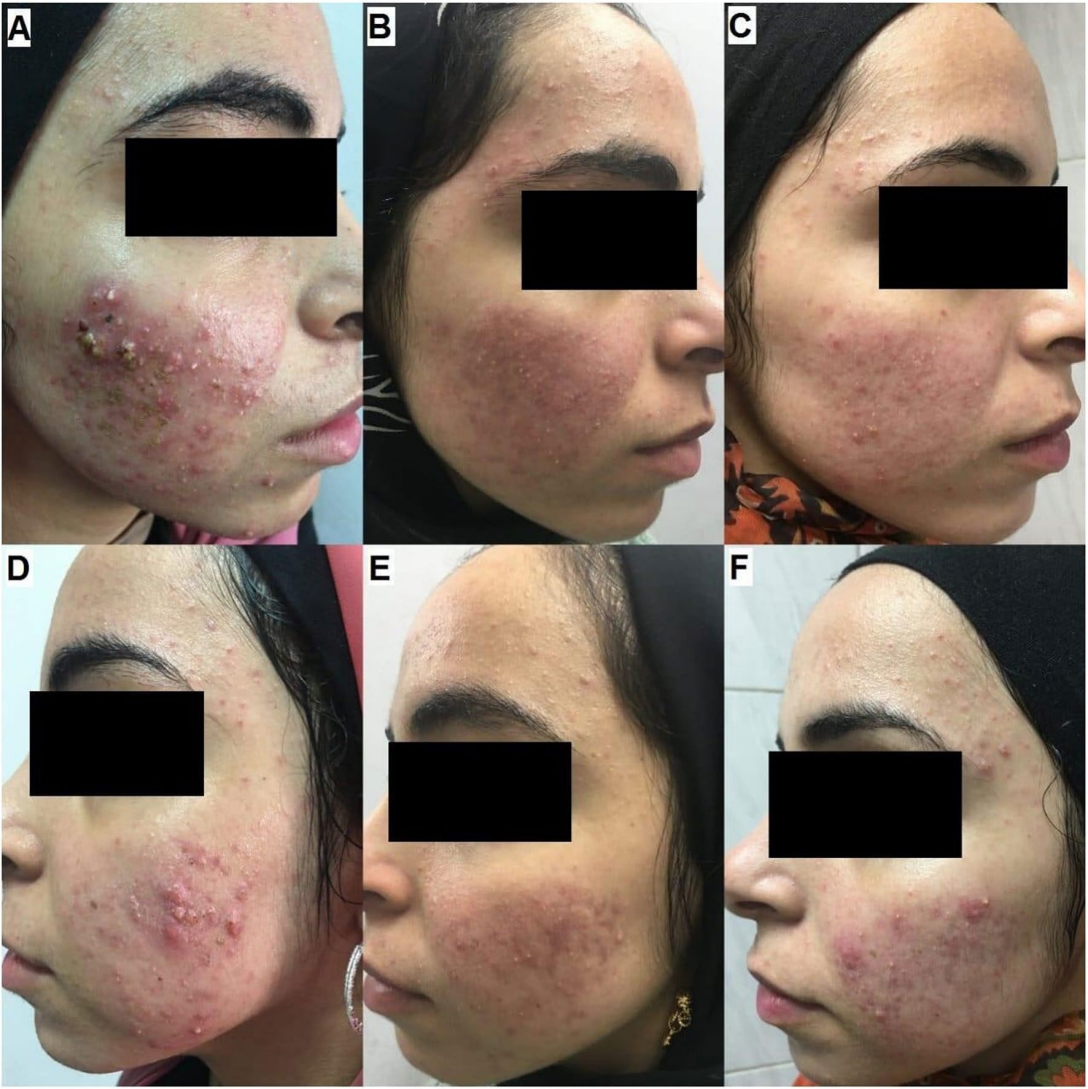
A 19-year-old female patient with very severe inflammatory acne (GEA scale was 5) received four treatment sessions. **A** Fractional laser side before treatment (GEAs 5, very severe). **B** Fractional side at the end of treatment (GEAs 1, almost clear). **C** Fractional side at a 3-month follow-up (GEAs 1, almost clear). **D** Nd-Yag laser side before treatment (GEAs 5, very severe). **E**. Nd-Yag side at the end of treatment (GEAs 1, almost clear). **F** Nd-Yag side at a 3-month follow-up (GEAs 2, mild)

**Fig. 4:**
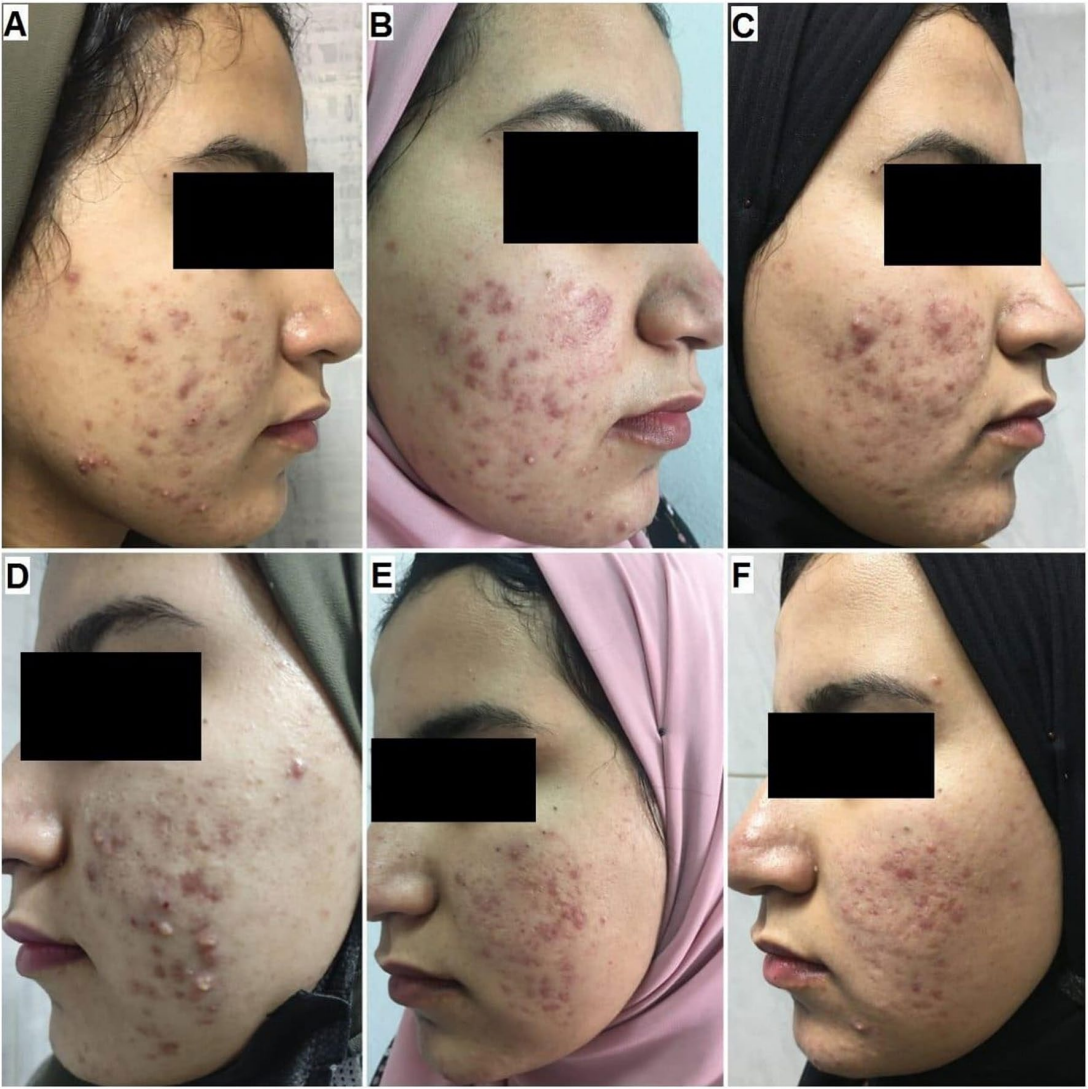
A 21-year old female patient with severe inflammatory acne (GEA scale was 4) received four treatment sessions. **A** Nd-Yag laser side before treatment (GEAs 4, severe). **B** Nd-Yag side at the end of treatment (GEAs 3, moderate). **C** Nd-Yag side at a 3-month follow-up (GEAs 3, moderate). **D** Fractional laser side before treatment (GEAs 4, severe). **E** Fractional side at the end of treatment (GEAs 3, moderate). **F** Fractional side at a 3-month follow-up (GEAs 3, moderate)

**Fig. 5:**
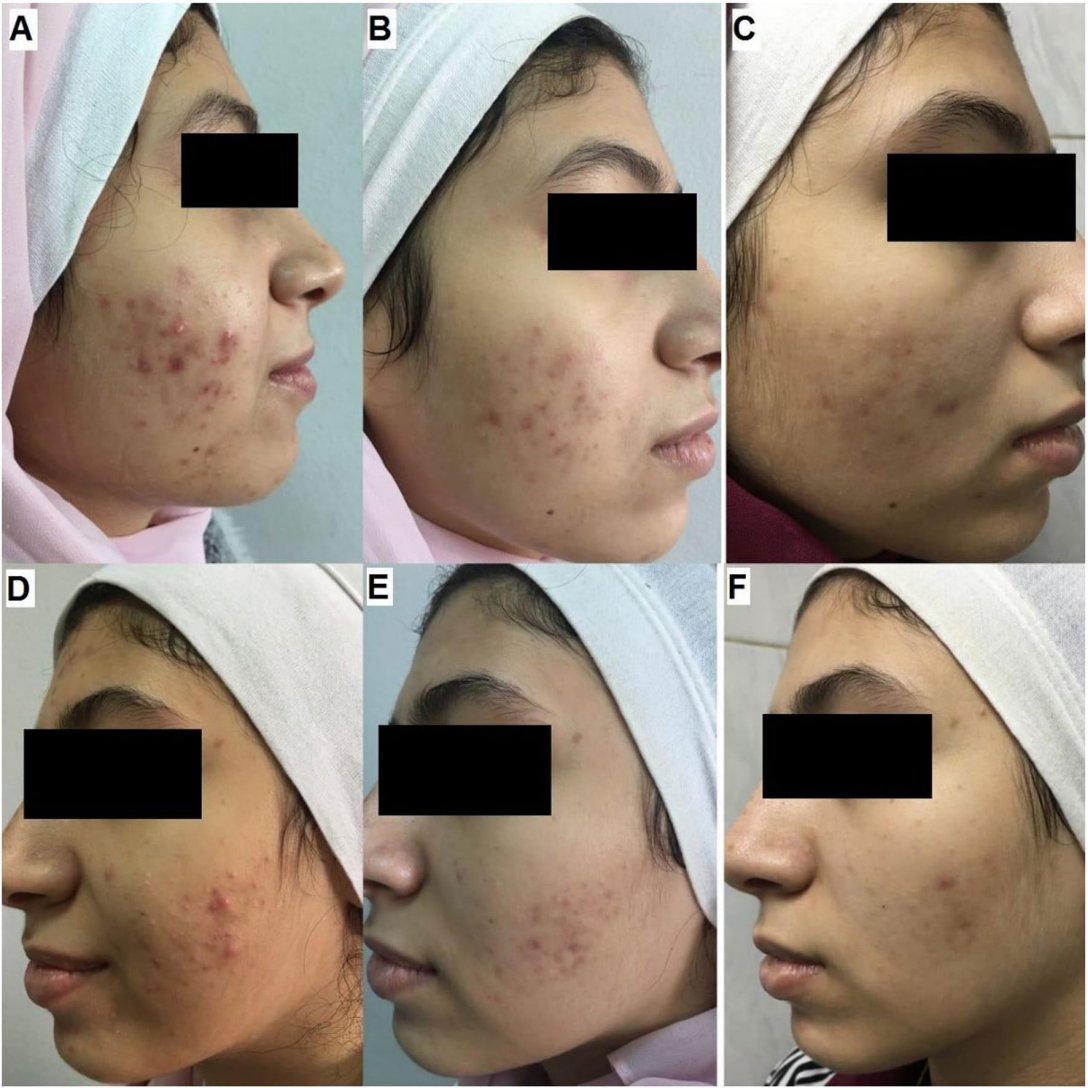
A 22-year-old female patient with moderate inflammatory acne (GEA scale was 3) received four treatment sessions. **A** Fractional laser side before treatment. **B** Fractional side at the end of treatment (GEAs 0, clear). **C** Fractional side at a 3-month follow-up (GEAs 0, clear). **D** Nd-Yag laser side before treatment (GEAs 3, moderate). **E** Nd-Yag side at the end of treatment (GEAs 0, clear). **F** Nd-Yag side at a 3-month follow-up (GEAs 0, clear)

Clinical photos of participants demonstrating successful treatment of acne by both modalities (Figs. 2, 3, 4, and 5).

A statistically significant higher satisfaction level and PGIG were observed in fractional Co2 treated side in comparison to Nd:YAG laser side with *P* = 0.004 and 0.001, respectively (Table 3). Participants were generally tolerated with the treatments; however, fractional CO_2_ laser was more painful than Nd:YAG laser with (*P* value = 0.000) (Table 3). There was no statistically significance between both modalities considering recurrence with (*P* value = 0.316) (Table 4).

**Table 3:** Comparison between fractional side and Nd-YAG side regarding patient’s satisfaction, pain level, and PGIG

		Fractional side	Nd-YAG side	Test value ‡	*P* value
		No. = 30	No. = 30		
Satisfaction level	Median (IQR)	8 (8 – 9)	7 (6 – 9)	−2.851	0.004
	Range	7 – 10	4 – 10		
Pain level	Median (IQR)	8 (7 – 9)	5.5 (4 – 6)	−4.838	0.000
	Range	5 – 10	0 – 10		
PGIG	Median (IQR)	6 (6 – 7)	6 (5 – 6)	−3.342	0.001
	Range	6 – 7	4 – 7		

*P* value > 0.05: non significant (NS); *P* value < 0.05: significant (S); *P* value < 0.01: highly significant (HS)

^‡^: Mann Whitney test

**Table 4:** Comparison between fractional side and Nd-YAG side regarding recurrence during follow-up period

Recurrence	Nd: YAG laser side	Fractional CO2 laser side	Test value	*P* value
No	23 (76.7%)	26 (86.7%)	1.002*	0.316
Yes	7 (23.3%)	4 (13.3%)		

*P* value > 0.05: non significant (NS); *P* value < 0.05: significant (S); *P* value < 0.01: highly significant (HS)

^*^: Chi-square test

Six patients had acne scars with the inflammatory acne; fractional CO_2_ laser-treated side evoked more improvement on the Goodman and Baron qualitative and quantitative acne scarring grading system in comparison to Nd-YAG laser side (*P* value < 0.001) (Table 5).

**Table 5:** Comparison between fractional CO2 laser side and Nd-YAG laser side regarding the Goodman and Baron qualitative and quantitative acne scarring grading system

		Fractional CO2 laser side	Nd-YAG laser side	Test value	*P* value
		No. = 6	No. = 6		
Qualitative grading					
Before	II	0 (0.0%)	1 (16.7%)	1.833	0.400
	III	5 (83.3%)	3 (50.0%)		
	IV	1 (16.7%)	2 (33.3%)		
3 months after	II	6 (100.0%)	1 (16.7%)	8.571	0.003
	III	0 (0.0%)	5 (83.3%)		
Improvement	Poor	0 (0.0%)	4 (66.7%)	6.286	0.043
	Good	5 (83.3%)	2 (33.3%)		
	Excellent	1 (16.7%)	0 (0.0%)		
Quantitative grading					
Before	Mean ± SD	15.50 ± 3.73	14.00 ± 3.85	0.686	0.508
	Range	12 – 21	10 – 19		
3 months after	Mean ± SD	4.83 ± 1.33	8.67 ± 3.27	–2.663	0.024
	Range	3 – 6	4 – 13		
*P* value		< 0.001 (HS)	0.002 (HS)		
% of improvement	Mean ± SD	68.67 ± 6.42	38.85 ± 14.30	4.658	0.001
	Range	57.14 – 75	18.75 – 60		
Reduction	Poor	0 (0.0%)	0 (0.0%)	6.000	0.048
	Minimal	0 (0.0%)	3 (50.0%)		
	Moderate	3 (50.0%)	3 (50.0%)		
	Good	3 (50.0%)	0 (0.0%)		

Fractional CO_2_ laser-treated side evoked more improvement on facial pore size in comparison to Nd-YAG laser side with *P* value (0.002) (Table 6).

**Table 6:** Comparison between fractional CO2 laser side and Nd-YAG laser side regarding improvement of facial pore size

Improvement	Fractional side		ND-YAG side		Test value	*P* value
	No	%	No	%		
No improvement	0	0.0%	0	20.0%	18.849	0.002
Mild improvement	3	10.0%	11	36.7%		
Moderated improvement	7	23.3%	15	50.0%		
Marked improvement	15	50.0%	4	13.3%		
Very significant improvement	5	16.7%	0	0.0%		

*P* value > 0.05: non significant (NS); *P* value < 0.05: significant (S); *P* value < 0.01: highly significant (HS)

^*^: Chi-square test

No adverse effects, such as post-laser hyperpigmentation, prolonged erythema (> 48 h), or post-laser hypopigmentation, were observed by the participants.

## Discussion

Efficacy of Nd:YAG laser to treat acne has been established in multiple studies [5, 6]. Fractional CO_2_ ablative laser resurfacing improves acne scarring and photoaging [18, 19]. However, there is limited evidence and few studies about the benefits of fractional CO_2_ lasers in treating active acne. This is the first study to compare a fractional CO_2_ laser with Nd:YAG laser in acne vulgaris. The split-face strategy was utilized to manage the contradictory factors that might have influenced the findings [5].

The current study demonstrated improvement of AV lesions by both Nd:YAG laser and fractional CO_2_ laser through a more significant reduction in GEAs score, and the mean inflammatory count in the fractional CO_2_ side than in the Nd:YAG side at the end of treatment; also, a reduction in the comedonal (noninflammatory lesions) was achieved but with no significant difference between the two modalities. Nonetheless, 3 months after the last session, the reduction of GEAs score, noninflammatory and inflammatory lesion numbers were significantly greater on the side treated with fractional CO2.

On Nd:YAG side, inflammatory and comedonal acne lesion numbers decreased by 81.41% and 61.70%, respectively, from baseline to a month after the final session.

Nd:YAG influences sebum output through sebaceous gland damage, normalizes follicular keratinization and corneocyte cohesion, upregulates TGF-β, and decreases the production of inflammatory cytokines, including interleukin-8, matrix metalloproteinase-9, nuclear factor kappa B, toll-like receptor (TLR)-2, and tumor necrosis factor-α [20].

It is thoughted that, the damaging effect of 1064-nm Nd:YAG on the dilated superficial vessel of inflammatory acne and the changes of cytokine release, including the upregulation of TGF-β and the downregulation of IL-8 and TLR-2, were associated with the improvement of inflammatory acne lesions [21–23]. The resolution of noninflammatory acne might be explained by the thermal destruction to sebaceous glands causing a decrease in sebum production [24].

The current results correlate with those of Chalermsuwiwattanakan et al. [5], who compared Nd:YAG laser to 595-nm pulsed dye laser in acne. They reported a significant decline in inflammatory lesions by 50.06%, from 5.76 ± 3.70 to 2.88 ± 2.86, and noninflammatory lesions by 15.95%, from 24.59 ± 15.36 to 20.67 ± 12.13 after three sessions of a 2-week interval.

Concerning the considerable improvement of the inflammatory lesions, these findings parallel our study findings with a substantial decline in noninflammatory lesions by 61.71%, which might be attributable to differences in patient demographics, number of sessions, and Nd:YAG parameters.

Similar to the present results, long-pulsed Nd:YAG laser 1064 nm demonstrated improvement percentage of inflammatory acne lesions of 70.42% after the third session of a 1-month interval therapy and increased to 85.72% following a 3-month follow-up, while the improvement percentage of noninflammatory acne lesions was 41.8% after treatment sessions and increased to 49.05% after 3 months of follow-up [6].

In addition, Monib et al. [21] observed a 65.7% improvement in inflammatory acne and a 44.0% improvement in noninflammatory acne following the third session at a 14-day interval using Nd:YAG.

It was evident that, similar to the present study, these trials demonstrated a more significant improvement in inflammatory lesions than in comedonal lesions. However, in the present study, the response rates were higher, which could be due to variations in participant features, device parameters and manufacturer, therapy session number, and time between sessions.

On the fractional CO_2_ laser side, inflammatory lesions showed a significant reduction after therapy, with a percentage of 87.94% ± 14.31, and a noninflammatory lesion, with a percentage of 78.06% ± 19.51.

The high improvement percentage regarding noninflammatory and inflammatory lesions on the fractional CO_2_ laser-treated side could be attributed to creating channels through the epidermis to the dermis, reducing the occlusion of the duct gland with transepidermal removal of sebum, coagulation of the channels’ nearby region, reducing inflammation and the vascular element [8]; thus, it creates significant residual thermal damage on the nearby tissues, which could induce protein denaturation, reduce bacteria in the follicle (P. acnes), and influence the gland [9, 25]. A cystic lesion seems to be an excellent target for a CO_2_ laser whose primary chromophore is water, and selective photothermolysis of the surrounding tissue could occur owing to heat diffusion within the cyst, as described by the theory of elongated selective photothermolysis for spherical structures [26].

A few studies have considered fractional CO_2_ laser use for treating acne. Seven Korean patients were the first to demonstrate a moderate (26–50%) to full (> 75%) reduction in acne lesions after two to three sessions of fractional CO_2_ laser therapy for inflammatory acne [27].

Pestoni Porvén et al. [7] detailed the successful treatment of two cases of microcystic and nodulocystic acne with only one/a single therapy with fractional CO_2_ laser in addition to topical retinoids and antibiotics.

In addition, Shin et al. [28] compared fractional CO_2_ laser versus fractional microneedle radiofrequency in acne patients and found that the mean decrease of papule and pustule counts was 54% and 41%, respectively, after 1–2 sessions of fractional CO_2_ laser, which agrees with our finding. In the current study, higher results were obtained which could be due to variations in participant features, fractional CO_2_ parameters, number of passes, laser device manufacturer, therapy session number, and time between sessions.

Adverse effects in this study such as erythema and edema were transient and progressively reduced immediately after treatment with the Nd:YAG laser and approximately 24 h following fractional CO_2_ laser therapy.

Fractional CO_2_ laser evoked a more significant improvement of facial pore size than Nd-YAG laser in the present study, where 20 cases (66.7%) exhibited a noteworthy to very substantial reduction in the size of their pores following fractional CO2 laser treatment. This can be explained by the effect of fractional CO2 on collagen synthesis [29, 30], as the rise in collagen production enhances dermal thickness, skin texture, and tone. In addition, fractional reticular cutaneous ablation results in collagen remodeling surrounding pores, which shrinks collagen fibers and tightens pores [31].

Our findings were marginally inferior to those of Eldeeb et al. [32], who showed more than 85% of patients responded with a marked to exceptional level to fractional CO2 laser therapy. While, our findings were better than those of Kown et al. [31], who examined the utilization of fractional CO2 laser on 32 Asian patients with enlarged facial pores. All patients showed moderate to significant improvement (26–75%), as reported previously.

Nd:YAG laser treatment resulted in moderate to significant enhancement of wide pores in 19 cases (63.3%). This can be attributed to the thermal or mechanical impacts of 1064-nm laser on fibroblasts, resulting in the production of novel elastin and collagen as well as collagen remodeling [33–35]. Similar findings were observed by Wattanakrai et al. [36] and Wang et al. [37], who observed reduced pores by an average of 32.9% and 21.7% respectively on the Nd:YAG laser side.

The present study involved six cases with acne scars in addition to active lesions; fractional CO_2_-laser treated side evoked a significant more improvement on the Goodman and Baron quantitative and qualitative acne scarring grading system in comparison to Nd-YAG laser side.

It is recognized that Nd:YAG lasers generate heat shock protein 70 and procollagen I from dispersed dendritic cells in the papillary and upper reticular dermis, resulting in collagen deposition in the papillary dermis [38].The current results correlate with previous researches [23, 39] who observed mild to moderate improvement in atrophic facial acne scars after sessions of long-pulsed Nd:YAG laser 1064 nm.

In the present study, the percent of improvement of acne scars was 68.67% after fractional CO_2_ therapy. Improvement of acne scars by fractional CO_2_ laser alone in our study was similarly evident in previous researches [40, 41].

## Conclusion

Fractional CO2 and Nd:YAG 1064-nm lasers are safe, tolerable, and highly effective therapeutic options for acne. However, fractional CO2 laser had a higher percent of improvement and patient’s satisfaction compared with long pulsed Nd:YAG. To our knowledge, this seems to be the first comparison between fractional CO2 laser and Nd:YAG laser in AV.

### Limitations

The small number of participants (*n* = 30) necessitates additional controlled researches to validate the use of fractional CO_2_ laser as a therapeutic option in active AV in a broader patient population.

### Future considerations

Encouraging researches on application of fractional CO_2_ laser (10,600 nm) for active acne vulgaris with increasing number of patients, longer follow-up, different age groups, different comparisons with other lasers or anti-acne drugs as a drug delivery laser for more favorable outcome, especially if other options are disabled, with psychologically affected patients, and who are demanding for effective and rapid treatment should be considered in the future.

## Data Availability

The data that support the findings of this study are available from the corresponding author upon reasonable request.
